# ICln: A New Regulator of Non-Erythroid 4.1R Localisation and Function

**DOI:** 10.1371/journal.pone.0108826

**Published:** 2014-10-08

**Authors:** Claudia Bazzini, Lorena Benedetti, Davide Civello, Chiara Zanoni, Valeria Rossetti, Davide Marchesi, Maria Lisa Garavaglia, Markus Paulmichl, Maura Francolini, Giuliano Meyer, Simona Rodighiero

**Affiliations:** 1 Department of Biosciences, University of Milan, Milan, Italy; 2 Department of Medical Biotechnology and Translational Medicine, University of Milan, Milan, Italy; 3 Fondazione Filarete for Biosciences and Innovation, Milan, Italy; 4 Pharmaceutical Sciences Department (DISFARM), University of Milan, Milan, Italy; 5 Institute of Pharmacology and Toxicology, Paracelsus Medical University, Salzburg, Austria; University of Vienna, Max F. Perutz Laboratories, Austria

## Abstract

To optimise the efficiency of cell machinery, cells can use the same protein (often called a hub protein) to participate in different cell functions by simply changing its target molecules. There are large data sets describing protein-protein interactions (“interactome”) but they frequently fail to consider the functional significance of the interactions themselves. We studied the interaction between two potential hub proteins, ICln and 4.1R (in the form of its two splicing variants 4.1R^80^ and 4.1R^135^), which are involved in such crucial cell functions as proliferation, RNA processing, cytoskeleton organisation and volume regulation. The sub-cellular localisation and role of native and chimeric 4.1R over-expressed proteins in human embryonic kidney (HEK) 293 cells were examined. ICln interacts with both 4.1R^80^ and 4.1R^135^ and its over-expression displaces 4.1R from the membrane regions, thus affecting 4.1R interaction with ß-actin. It was found that 4.1R^80^ and 4.1R^135^ are differently involved in regulating the swelling activated anion current (I_Cl,swell_) upon hypotonic shock, a condition under which both isoforms are dislocated from the membrane region and thus contribute to I_Cl,swell_ current regulation. Both 4.1R isoforms are also differently involved in regulating cell morphology, and ICln counteracts their effects. The findings of this study confirm that 4.1R plays a role in cell volume regulation and cell morphology and indicate that ICln is a new negative regulator of 4.1R functions.

## Introduction

ICln [1] is a ubiquitously expressed and highly conserved [2] 26 kDa protein, whose knock-out in mice is lethal [3]. This is consistent with its essential role in one or more basic cell processes, and it has therefore been proposed to be a connector hub [4] of such diverse cell functions as ion permeation [5], cytoskeletal organisation [6]–[9] and RNA processing [10].

It mainly localises in the cytoplasm, but it is also present in the nucleus [6] and in membrane regions upon hypotonic stimulation [11]. In the cytoplasm, ICln acts as a chaperone assembly factor that is involved in the formation of small nuclear ribonucleoproteins (snRNPs) [12], [13]. Its hypotonically induced shift towards the membrane area plays a role in activating the anion current that is activated upon cell swelling (I_Cl,swell_) [11], [14], [15], but the mechanism underlying this effect is unclear. The ICln binding/unbinding regulation of cytoskeletal proteins may be an important step in the modulation of channel/transporter function because ICln interacts with actin [6], [7] and, in renal collecting duct cells, this interaction increases after cell swelling [14].

ICln also interacts with the multifunctional 4.1R cytoskeletal protein [8], [16] but the functional role of this interaction has not yet been investigated. The finding that 4.1R-null mouse erythrocytes are characterised by cell dehydration due to the hyperactivity of NHE1 [17], [18], the ubiquitous Na^+^/H^+^ exchanger that is activated by cell shrinkage and inhibited by cell swelling [19], [20], indicates that 4.1R protein plays a role in cell volume regulation.

Endogenous and transiently transfected 4.1R isoforms have been detected in the cytoplasm, nucleus and membrane regions of nucleated cells [21]–[25]. The presence of 4.1R proteins in membrane regions is crucial as they regulate the abundance and function of transmembrane structural proteins [26], receptors [17], [25], [27], transporters [28], [29] and channels [30] by acting as membrane hub proteins [31].

In erythroid and non-erythroid cells, multiple isoforms of 4.1R are often simultaneously expressed as a result of three distinct mechanisms: the alternative splicing of pre-mRNA [32]–[35]; the presence of an internal ribosome entry site (IRES) that allows the translation of different isoforms from different translation-initiation codons (ATG1, ATG2 and probably ATG3) [22], [36] from a single mRNA [37]; and post-translational modifications [24], [38]–[41]. The first two mechanisms produce 4.1R isoforms with different exon compositions: the 135 kD (4.1R^135^), 80 kD (4.1R^80^) and 60 kD isoforms (4.1R^60^). All isoforms share highly conserved domains: the 4.1 and ezrin/radixin/moesin (FERM) domain, the spectrin-actin binding domain (SABD), and the C-terminal domain (CTD).

The selective expression of alternatively spliced mRNA seems to be developmentally regulated during cell maturation/differentiation [22], [33], [42]–[44], and influences 4.1R intracellular localisation and function [29],[35],[36],[45]. However, the functional differences between these isoforms and the functional need to express so many apparently redundant proteins have not yet been fully elucidated. For this reason, identifying the cell mechanisms responsible for the intracellular localisation of 4.1R and its compartmentalised interactions may therefore also have considerable implications for the study of its functions.

We examined the intracellular localisation and function of 4.1R^80^ and 4.1R^135^ in a nucleated human cell line under basal conditions and during hypotonic cell swelling. The only difference between the two isoforms is the presence of the 209 N-terminal amino acids of the headpiece domain coded by AUG-1 in 4.1R^135^. ICln interacts with both isoforms and, when over-expressed, promotes the displacement of 4.1R from the membrane region and decreases the interaction between 4.1R and subcortical F-actin.

The two isoforms differently affect I_Cl,swell_ activation upon cell swelling and, during hypotonic stimulation, the amount of 4.1R in the membrane region decreases. Moreover, 4.1R over-expression induces cell spreading and the emission of filopodia, an effect that can be reverted by ICln over-expression.

Our findings strongly suggest a new role for ICln as a regulator of 4.1R localisation and function, and confirm that 4.1R plays a role in cell volume regulation.

## Materials and Methods

### Plasmids and transfection

All of the DNA constructs were confirmed by sequencing. The cDNAs corresponding to the human open reading frame (ORF) of 4.1R^80^ and 4.1R^135^ were obtained by means of RT-PCR from HEK (human embryonic kidney) cells (LGC Standards S.r.l., Italy). The only difference between the two DNAs was the presence (4.1R^135^) or absence (4.1R^80^) of the 209 N-terminal amino acids of the headpiece (HP) domain. The exon organisation was the same as that reported for isoforms 4.1R^135^ and 4.1R^80^ in erythroid cells [22]: i.e. both isoforms lacked exons 13–14 (human gene EBP41, NCBI Reference Sequence:XP_005245821 for 4.1R^135^ and GenBank refrence sequence AAH96105.2 for 4.1R^80^). The 4.1R^80^ and 4.1R^135^ cDNAs were sub-cloned into pEYFP-C1 vectors (Y-4.1R^80^, Y-4.1R^135^) (Clontech, Mountain View, CA, USA) in order to express YFP-tagged proteins respectively C-terminally (C-t) or N-terminally (N-t), and in the pIRES2-EGFP (4.1R^80/135^-IRES-GFP) bicistronic vector (Clontech), in order to express the chosen and the fluorescent protein as two distinct polypeptides. All vector variants expressing 4.1R^135^ were obtained by additionally mutating the ATG2 codon in exon 4 into GTG, using the Quickchange Site-Directed Mutagenesis kit (Agilent Technologies Italia S.p.A., Milan, Italy), to prevent the production of 4.1R^80^ from 4.1R^135^, promoted by the presence of an internal ribosome entry site (IRES) between ATG1 and ATG2 [37]. Similarly, pECFP-C1 containing the ORF of hICln (human gene CLNS1A, NCBI Reference Sequence: NP_001284.1), C-ICln, or the ORF of a truncated ICln (aa 1-102, C-IClnT), were used to express CFP-ICln chimeras. The ORFs for ICln was also inserted in the pFLAG CMV4 vector (Sigma-Aldrich S.r.l., Milan, Italy) in order to obtain the FLAG C-t tagged ICln protein (FLAG-ICln), and in the pIRES2-dsREDexpress (ICln-IRES-dsRED) bicistronic vector (Clontech). The cDNA for the human β-actin (human gene ACTB, GenBank reference sequence: BC016045.1) ORF was inserted into the pECFP-C1 vector in order to obtain the C-βactin vector. The ptdTomato-N1 vector (Clontech) was used in the siRNA experiments to express the Tomato protein; the vector is designed with two copies of the Tomato coding region linked together to allow intramolecular dimerization (tdTomato).

HEK cells were transiently transfected 24 hours post-seeding, and then used for experiments 24 or 48 hours post-transfection depending on the experimental protocol. In the co-transfection experiments, each vector was equimolar in the transfection mix.

### Cell culture

Human embryonic kidney (HEK) 293T cells were cultured in Eagle's Minimum Essential Medium (EMEM, Sigma, Italy) supplemented with 10% Fetal Bovine Serum (FBS, Lonza S.r.l., Milan, Italy), 1 mM sodium pyruvate, 2 mM L-glutamine, 0.1 mM non essential aminoacids, 100 U/ml penicillin, 100 µg/ml streptomycin (Sigma, Italy). Cell cultures were maintained at 37°C with 5% CO2 and passaged every 3–4 days.

### Patch-clamp experiments

The patch-clamp experiments were performed in whole-cell configuration using HEK cells transiently transfected with the bicistronic vector pIRES2-EGFP expressing a 4.1R isoform (pIRES2-EGFP-4.1R^135^, pIRES2-EGFP-4.1R^80^). The pIRES2-EGFP vector, which expresses only EGFP, was used as control. The pipette solution contained (mM) 125 CsCl, 11 EGTA, 5 MgCl_2_, 2 Mg-ATP, 50 raffinose and 10 HEPES (pH 7.2, 336 mOsm); the hypertonic bath solution contained (mM) 125 NaCl, 2.5 CaCl_2_, 2.5 MgCl_2_, 100 mannitol and 10 HEPES (pH 7.4, 375 mOsm), and the hypotonic bath solution contained (mM) 125 NaCl, 2.5 CaCl_2_, 2.5 MgCl_2_ and 10 HEPES (pH 7.4, 275 mOsm). All of the experiments were performed at room temperature.

The pipettes were pulled from borosilicate glass capillaries and had a resistance of 3–5 MΩ after fire polishing. Seal resistances were typically between 3 and 10 GΩ. The currents were recorded using an EPC9 amplifier (HEKA, Lambrecht/Pfalz Germany) and low-pass filtered at 2.9 kHz. The data were analysed using Pulse/Pulsefit software (HEKA). The bath was grounded by means of an Ag/AgCl electrode immersed in the bath solution. The GFP-positive cells were identified immediately before cell patching using a fluorescence-equipped inverted microscope (ECLIPSE TS 100, Nikon Instruments S.p.A. Campi Bisenzio, Florence, Italy). Pipette and whole-cell capacitance and series resistance compensations were made before the recording. I-V relationships were obtained with a step-protocol, by averaging the currents generated by pulsing (0.5 s) from -100 mV to +100 mV, with step increments of 20 mV; the holding voltage between pulses was 0 mV. The currents were normalised to cell membrane capacitance, and expressed as current density (pA/pF). In order to construct time courses of current activation, current amplitude was measured at a constant potential of +40 mV every 10 s until 10 min after hypotonic replacement. Membrane capacitance did not change during each experiment, and was not affected by the clone transfections.

### FRET

The 4.1R/ICln interaction FRET experiments were performed 24 hours after transfection using living cells kept at 37°C with CFP (cyan fluorescent protein) as the donor and YFP (yellow fluorescent protein) as the acceptor molecule. The experiments were carried out using cells kept in a slightly hypertonic extracellular solution (in mM: NaCl 90, KCl 5, CaCl_2_ 2, MgCl_2_ 2, glucose 5, HEPES 10 and mannitol 100, 317 mOsm, pH 7.4), or after exposure to a hypotonic extracellular solution obtained by omitting mannitol from the hypertonic solution.

In the case of the 4.1R/ß-actin interaction FRET experiments, the cells were fixed in 4% paraformaldehyde (PFA) in PBS (in mM: 137 NaCl, 2.7 KCl, 1.8 KH_2_PO_4_, 10 Na_2_HPO_4_, pH 7.4) for 10 min, and kept in PBS during the confocal acquisitions.

The sensitised emission (Fsen) and NFRET indices (Fsen normalised by the acceptor emission) were calculated according to [46].

FRET efficiency (FRETeff) was measured using acceptor photobleaching [47]. The images were acquired by means of a Leica TCS SP5 confocal microscope (Leica Microsystems GmbH, Wetzlar Germany). In order to avoid the possible diffusion of fluorescent protein in and out of the region of interest (ROI) during the photobleaching of live cells, the whole of the cell under examination was bleached. The images were acquired using an HCX PL APO 63x/1.4 OIL objective (Leica Microsystems GmbH) and a scan speed of 700 Hz.

FRETeff was then evaluated using the FRETcalc ImageJ plugin [48] as previously reported [49].

### Confocal microscopy

The images of over-expressed YFP-tagged 4.1R and CFP-tagged ICln were acquired 24 hours post-transfection using a confocal microscope equipped with an HCX PL APO 40x/1.25 OIL objective (Leica Microsystems GmbH). During the acquisition, the living HEK cells were kept at 37°C in DPBS (PBS supplemented with 1 mM CaCl_2_, 0.5 mM MgCl_2_, 25 mM glucose, pH 7.4).

The confocal imaging of the co-localisation experiments involved living cells kept at 37°C in the microscope incubator 24 hours after transfection. CFP-mem (Cm) was used as a membrane marker, and Pearson and Manders coefficients were calculated from the whole-cell Z-stacks acquired using a Leica TCS SP5 confocal microscope equipped with a resonant scanner and an HCX PL APO 63x/1.4 OIL objective (scan speed 8000 Hz, pixel size 98.41×98.41×250 nm). The same fields were acquired in a hypertonic extracellular solution (317 mOsm), and after 5 and 10 minutes of hypotonic substitution (217 mOsm). The co-localisation analyses were made using the ImageJ JACoP plug-in [50] on the entire stacks after the application of a filter (Gaussian Blur) in order to remove noise. To select the fluorescence signal associated with the plasma membrane, appropriate thresholds for each channel were applied and kept constant throughout the analysis of each cell (hyper, hypo 5′, hypo 10′).

### STED microscopy

The YFP signal of cells over-expressing a membrane marker (YFP-mem, Clontech) and the 4.1R^135^ protein was acquired using the confocal or gated-STED module of a Leica TCS SP8 microscope (Leica Microsystems GmbH, Wetzlar, Germany) equipped with an HCX PL APO 100x/1.4 OIL STED ORANGE objective, a white light laser (WLL) source, and a 592 nm depletion laser. The images were acquired using hybrid detectors with a pixel size of 20.7 nm and, in the case of g-STED, a time-gate between 1.5 and 6.5 nsec.

### Immunofluorescence

HEK cells seeded on glass coverslips were fixed with 3% paraformaldehyde in PBS and permeabilized with PBS containing 0.1% Triton X-100 and 3 mM MgCl_2_. Non-specific binding was blocked by means of 3% BSA in PBS. The cells were then incubated in the presence of a rabbit anti-4.1R primary antibody (EPB41, Sigma-Aldrich), 1∶400 dilution at 4°C overnight, followed by an Alexa 555 donkey anti-rabbit antibody (1∶200; Jackson ImmunoResearch Europe Ltd., Suffolk, UK). The coverslips were mounted in 90% glycerol/PBS, and acquired using a Leica TCS SPE AOBS confocal microscope equipped with an ACS APO 40x/1.15 OIL objective (Leica Microsystems GmbH). In the case of transfected cells, the samples were prepared 24 hours after transfection.

In the case of the immunofluorescence experiments with siRNA transfected HEK cells, ICln and 4.1R were separately immunolabelled in different specimens, to avoid the cross-reactivity of the secondary antibody, since both primary antibodies were raised in rabbit. Anti-rabbit Alexa 488 (Invitrogen) was used (1∶200 dilution) as secondary antibody in both cases. The same acquisition parameters of the Alexa 488 signal were used both for ICln siRNA and control siRNA samples. In the case of ICln immunolabelling, cells were fixed with 3% paraformaldehyde in PBS and permeabilized with PBS containing 0.1% Triton X-100 and 3 mM MgCl_2_. Non-specific binding was blocked by means of 3% BSA in PBS. The cells were then incubated with the anti-ICln antibody (1∶100 dilution) for 2 hours at room temperature, followed by the secondary antibody. The coverslips were mounted in 90% glycerol/PBS, and images were acquired using a Leica TCS SP5 AOBS confocal microscope equipped with an HCX PL APO 63x/1.4 OIL objective (Leica Microsystems GmbH). DNA was stained with DAPI (4′,6-diamidino-2-phenylindole).

### Correlative light-scanning electron microscopy (CLEM)

HEK cells were transfected with the following plasmids: EGFP-IRES (control), 4.1R^135^-IRES-EGFP, 4.1R^80^-IRES-EGFP, 4.1R^135^-IRES-EGFP and ICln-IRES-DsRED, 4.1R^80^-IRES-EGFP and ICln-IRES-DsRED, ICln-IRES-DsRED. Twenty-four hours post-transfection, the cells were seeded on micro-patterned Aclar discs functionalised with Poly-L-Lysine. The pattern, which consisted of an asymmetrical mesh of about 140 µm squares, was sculpted on Aclar film using the pulsed laser of a micro-dissecting microscope (Leica Microsystems GmbH) [51]. The coordinates allowed the identification of the same cell by both fluorescence microscopy and SEM.

The confocal images were acquired 48 hours post-transfection using an HCX PL FLUOTAR 20x/0.5 objective (Leica Microsystems GmbH). After image acquisitions, the cells were fixed with glutaraldehyde 1.2% in NaCacodylate 0.1 M for 1 h, and then post-fixed with 1% osmium tetroxide (OsO_4_) in NaCacodylate 0.1 M for 1 h. The samples were gradually dehydrated by means of an ethanol series, dried using a critical point drier (Emitech K850, Quorum Technologies Ltd, East Sussex, UK), and sputtered with gold (Polaron E5100 Sputter Coater, Quorum Technologies Ltd.) before the transfected cells were imaged by means of SEM (Zeiss Sigma Field Emission Scanning Electron Microscope, 1 kV, SE2 detector). The images acquired from each specimen in three independent experiments were used to measure the area, number and length of filopodia by means of ImageJ software.

### Protein preparations

#### Total membrane proteins

Twenty-four hours after transfection, the cells were collected by scraping, pelleted, resuspended in PBS in the presence of a complete EDTA-free protease inhibitor cocktail (Roche Diagnostics S.p.A., Milan, Italy), followed by repeated passages through a 29G needle. Total membrane proteins were extracted as described in [54], and protein concentrations were measured by means of a Bradford assay. The reliability of membrane separation was verified by confirming the enrichment of cadherin (a plasma membrane marker) in the plasma membrane fraction and comparing it with GAPDH, a cytosolic marker (data not shown).

In the case of the experiments using cells exposed to a hypotonic challenge, the cells were exposed for 15 min to a slightly hypertonic solution (in mM: NaCl 90, KCl 5, CaCl_2_ 2, MgCl_2_ 2, glucose 5, HEPES 10 and mannitol 100, 317 mOsm, pH 7.4) or hypotonic solution (obtained by omitting mannitol from the hypertonic solution), before being harvested and lysed.

#### Total protein extracts

The cells were lysed in Tris lysis buffer (20 mM Tris-HCl, 150 mM NaCl, 1 mM EDTA, 1% NP40, Roche Diagnostics complete EDTA-free protease inhibitor cocktail, pH 7) and snap-frozen in liquid nitrogen. The lysate was spun at 4500 g for 5 minutes, and then the supernatant was saved and stored at −80°C until use. Protein concentration was quantified by means of a Bradford assay.

### Western blotting

All of the protein extracts were heated at 99°C for 5 minutes in SDS-PAGE solubilising buffer (58 mmol/L Tris HCl, 10% glycerol, 2% SDS, 0.004% bromophenol blue, pH 6.8) containing 7.5% dithiothreitol. The proteins were separated by means of SDS-PAGE electrophoresis on a 10% polyacrylamide gel, and transferred to a PVDF membrane. After blocking, the membrane was incubated with anti-ICln [5], anti-actin I-19 (Santa Cruz Biotechnology, Dallas, Texas, USA), anti 4.1R C-16 (Santa Cruz Biotechnology) or anti-4.1R EPB41 (Sigma-Aldrich), anti-EGFP (Clontech), monoclonal anti-GAPDH (clone GAPDH-71.1, Sigma-Aldrich), anti-pan cadherin ABT35 (Abcam plc, Cambridge, UK), or anti-FLAG M2 antibody (Sigma-Aldrich), diluted in the blocking buffer at 4°C overnight, followed by several washes, and then by the secondary HRP-conjugated antibody. The Immobilon ECL system (Millipore S.p.A., Vimodrone, Italy) was used for detection.

The PVDF membrane was always stained using the amido black staining procedure in order to assess the efficiency of protein transfer and verify equal loading.

The bands were densitometrically analysed using the ImageJ software.

### Co-immunoprecipitation (co-IP)

#### FLAG-ICln co-IP

HEK cells co-transfected with pFLAG-ICln (or pFLAG-BAP [Sigma-Aldrich] in the case of controls) and 4.1R-Y or Y-4.1R chimeras, were lysed in Tris lysis buffer (25 mM Tris pH 8, 150 mM NaCl, 10% glycerol, Roche Diagnostics complete EDTA-free protease inhibitor cocktail, 0.5% Triton X-100), the cell debris were pelleted at 4500 g for 10 min (4°C), and the supernatants (2 mg) were immunoprecipitated using 100 µl of the anti-FLAG M2 affinity gel, a purified murine IgG1 anti-FLAG antibody covalently attached to agarose beads (Sigma-Aldrich). The bound protein complexes were eluted in the presence of enriched 130 µg/mL FLAG peptide (Sigma Aldrich) in PBS buffer in 40 µL aliquots, run on SDS-PAGE, and revealed by means of Western blotting using anti-4.1R (16-C, Santa Cruz Biotechnology) and anti-FLAG M2 antibodies (Sigma-Aldrich).

#### Actin co-IP

HEK cells transfected with 4.1R-IRES-EGFP vectors (alone or co-transfected with C-ICln or CFP) were lysed in CHAPS binding buffer (25 mM HEPES, 150 mM NaCl, 1 mM MgCl_2_, Roche Diagnostics complete EDTA-free protease inhibitor cocktail, and 0.5% CHAPS). After repeated syringing through a 20 gauge needle, the cell debris were pelleted at 4500 g for 10 min (4°C), and the supernatants (2 mg) were incubated at 4°C overnight with 40 µL of agarose-bound actin (I-19) AC goat Ig-G (0.5 µg/µL; Santa Cruz Biotechnology) or goat IgG-AC – agarose (sc-2346; Santa Cruz Biotechnology) in the case of controls. The bound protein complexes were eluted by resuspending the resin in 40 µL of 2X SDS-PAGE solubilising buffer, boiled for 5 min, and pelleted at 10000 g for 1 min. The supernatants were assayed (21 µL) by means of Western blotting using anti 4.1R 16-C and anti-actin I-19 antibodies.

### siRNA transfection

Scrambled siRNA (negative control) and validated ICln siRNA were purchased from Invitrogen. siRNAs were co-transfected with the ptdTOMATO-N1 vector (10∶1 w/w) into HEK cells by using Lipofectamine 3000 (Invitrogen), according to manufacturer instruction. Cells were used for western blot or immunofluorescence experiments 48 hours after transfection.

### Statistics

The data are expressed as mean values ± standard error of the mean. The differences between two groups were assessed using a two-tailed Student's t-test, and the differences among three or more groups were assessed using one-way ANOVA (analysis of variance) with Bonferroni's or Dunnet's multiple comparison post-test. The groups were considered significantly different when at least a 95% confidence level was obtained (p<0.05).

## Results

### ICln interacts with YFP-tagged 4.1R^80^ and 4.1R^135^ in HEK cells

**Figure 1:**
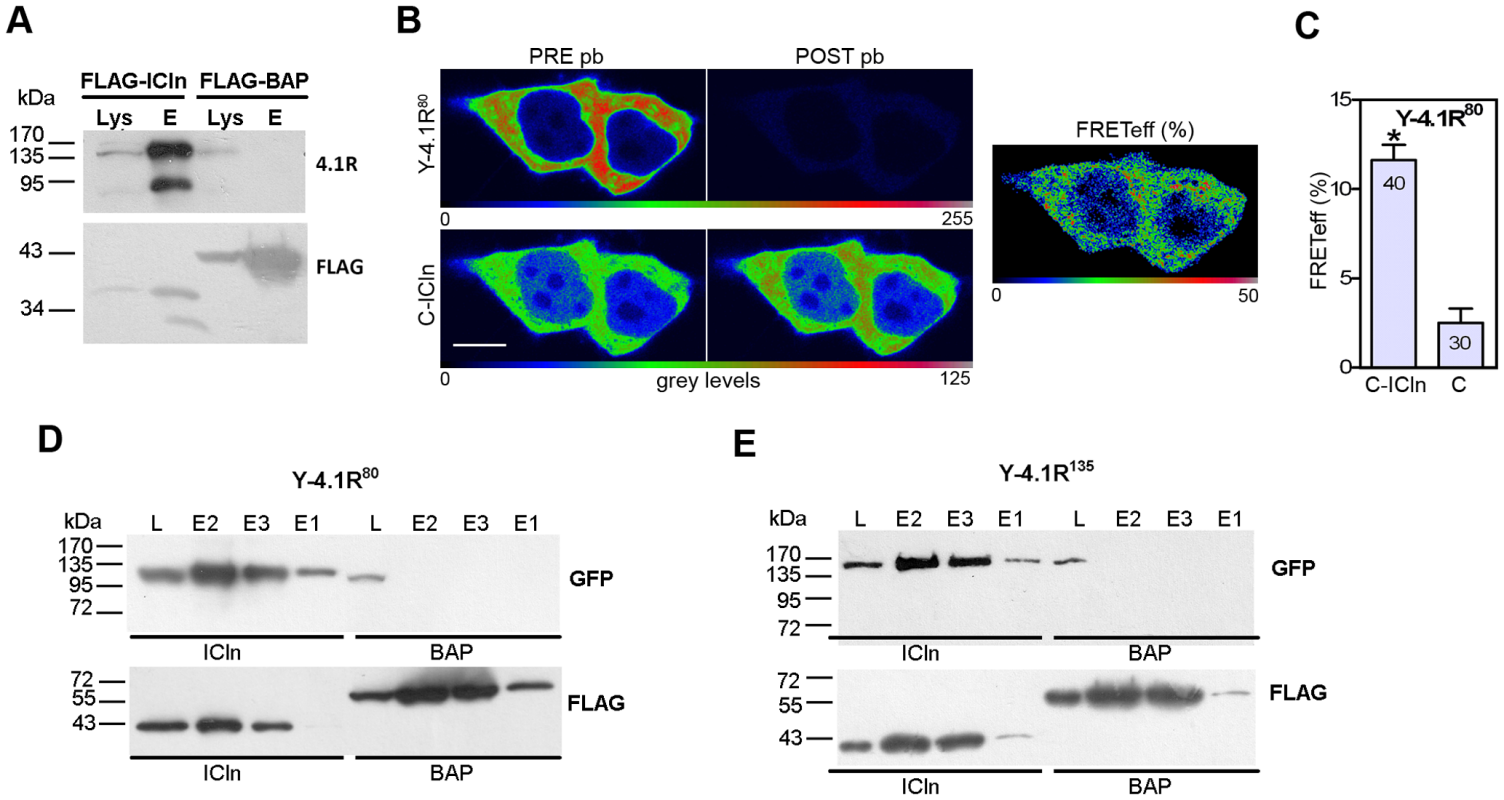
4.1R^80/135^ and ICln interactions in HEK cells: co-immunoprecipitation and FRET. (A) Co-immuprecipitation of FLAG-ICln and endogenous 4.1R in HEK cells. Anti-4.1R (*4.1R*) and anti-FLAG (*FLAG*) were respectively used to detect 4.1R and FLAGed proteins. Western blot showing immunoprecipitation of 4.1R with Flag-ICln, but not FLAG-BAP (control). (B) Images of an acceptor photobleaching FRET experiment using living cells over-expressing Y-4.1R^80^ and C-ICln. Pre-photobleaching (PRE pb) and post-photobleaching (POST pb) images are shown. Scale bar: 10 µm. (C) Quantification of FRET experiments with CFP-tagged ICln and YFP-tagged 4.1R^80^. The mean FRETeff ± SEM is plotted (*p<0.05 for Y-4.1R^80^+C-ICln *vs* Y-4.1R^80^+C; one-way ANOVA). The numbers inside the bars represent the number of cells analysed from at least 3 independent experiments. (D) Co-immunoprecipitation of Y-4.1R^80^ or (E) Y-4.1R^135^ with FLAG-ICln (*ICln*) or FLAG-tagged bovine alkaline peroxidase (*BAP*, control). The HEK cells were co-transfected with C-terminally FLAGed ICln and Y-4.1R^80^ or Y-4.1R^135^. FLAG-ICln was immunopurified using an anti-FLAG antibody. The 4.1R signal (anti-GFP antibody) and FLAG signal (anti-FLAG antibody) in cell lysates (L), and three sequential 40 µl eluates (E1-E3) are shown for all conditions.

In HEK cells, both low molecular weight (LMW) or high molecular weight (HMW) native 4.1R isoforms co-immunoprecipitated with the transfected C-terminally flagged ICln (FLAG-ICln) (Fig. 1A).

We used FRET studies to investigate the *in vivo* sub-cellular localisation of the 4.1R/ICln interaction, and the specific relationship between ICln and 80 or 135 kDa isoforms, using YFP-tagged 4.1R (Y-4.1R^80^ and Y-4.1R^135^) and CFP-tagged ICln (C-ICln). In comparison with the control C/Y-4.1R^80^, the C-ICln/Y-4.1R^80^ pair showed a statistically significant FRET signal (Figs. 1B and 1C); there was no significant FRET signal with the other FRET pair, Y-4.1R^135^/C-ICln (FRETeff C-ICln/Y-4.1R^135^ = 1.77±0.36, n = 26; FRETeff C/Y-4.1R^135^ = 0.83±0.38, n = 24, ns, *t*-test). The FRETeff calculated for Y-4.1R^80^ and a mutated C-ICln (C-IClnT) lacking the 4.1R binding site [8], was not different from the control (FRETeff C-IClnT/Y-41R^80^ = 4.16±0.89, n = 20; FRETeff C/Y-41R^80^ = 2.42±0.89, n = 10, ns, *t*-test), thus confirming the specificity of the interaction between Y-4.1R^80^ and C-ICln.

We used co-immunopreciptation experiments to verify the possibility of a 4.1R^135^/ICln interaction further (Figs. 1D and 1E). HEK cells were co-transfected with a C-terminally flagged ICln (FLAG-ICln) and the same 4.1R chimeras as those used in the FRET experiments. Both the 4.1R fusion proteins strongly immunoprecipitated with FLAG-ICln, thus suggesting that the unfavourable position of the fluorophores might be the main cause of the low FRET signals of Y-4.1R^135^.

### ICln over-expression in HEK cells inhibits 4.1R membrane localisation

**Figure 2:**
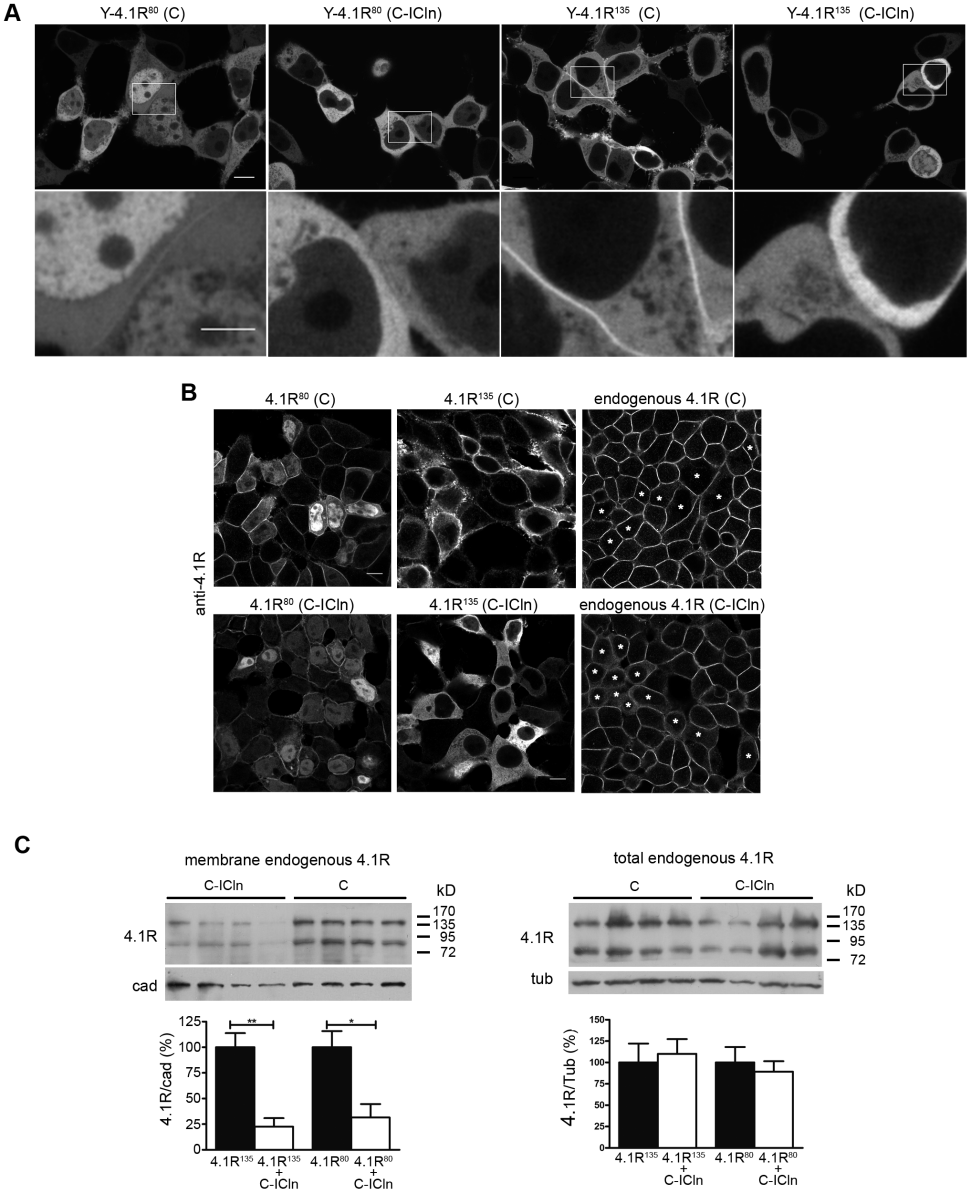
ICln over-expression affects 4.1R membrane localisation. (A) The images in the first row show the intracellular localisation of the indicated proteins (single confocal planes); the unshown co-transfected protein is indicated in brackets. The images in the second row are enlargements of the insets indicated in the first row images. (B) Exemplificative images of HEK cells co-transfected with GFP-IRES-4.1R^80^ (*4.1R^80^*) or GFP-IRES-4.1R^135^ (*4.1R^135^*) and CFP (*C*) or CFP-ICln (*C-ICln*) vectors. The samples were immunolabelled with an anti-4.1R antibody to visualise the 4.1R signal. In the panels showing the endogenous 4.1R signal, the asterisks indicate the CFP or C-ICln transfected cells. Scale bar 10 µm. (C) Effect of ICln on endogenous 4.1R membrane localisation: Western blot of total membrane protein extracts (*left*) and total endogenous 4.1R (*right*) from HEK cells transfected with C-ICln or C (control). The histograms show the mean OD value of the 4.1R signal normalised for the corresponding cadherin (*left*) or tubulin (*right*) signal (n = 4). The values are percentages of the control. **p<0.01; *p<0.05.

Both 4.1R variants contain exon 16, which is essential for the interaction with actin/spectrin [52] and nuclear targeting [36], and exon 5, which is involved in membrane binding [53] and nuclear export [45]. The localisation of both the chimeric (Fig. 2A) and native (Fig. 2B) 4.1R isoforms was consistent with the role of the two exons insofar as the nuclear localisation of 4.1R^135^ was reduced, which is in line with the reported inhibition of nuclear targeting exerted by the HP region [45].

Confocal imaging of HEK cells over-expressing YFP-tagged 4.1R unequivocally showed that C-ICln inhibited the membrane association of both Y-4.1R^80^ and Y-4.1R^135^ (Fig. 2A). The reduced membrane localisation of both proteins was accompanied by a cytoplasmic accumulation of 4.1R. This ICln-related effect was observed regardless of the cell confluence degree, when the untagged 4.1R proteins were over-expressed and labelled with the anti-4.1R antibody, and when endogenous 4.1R was visualised (Fig. 2B).

Western blot quantification showed that the membrane-bound pool of both endogenous 4.1R isoforms was statistically decreased by C-ICln over-expression (Fig. 2C). No significant effect was detected in the case of cadherin, which was used as internal control for the normalization of 4.1R signals in the quantitative analysis. The Western blot experiments on total protein preparations indicated that ICln did not significantly alter the global level of 4.1R expression (Fig. 2C).

**Figure 3:**
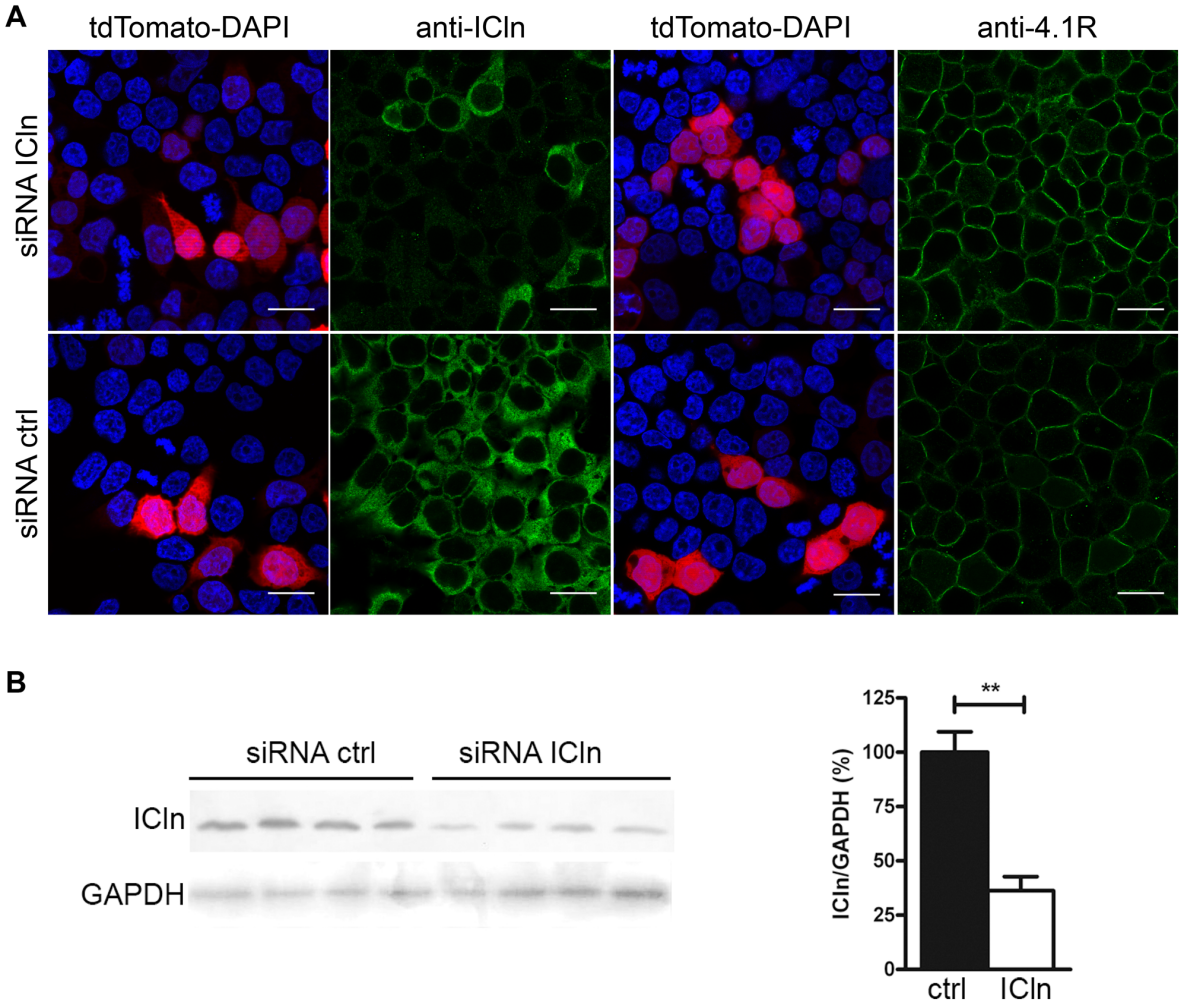
Downregulation of ICln by siRNA and 4.1R membrane localisation. (A) Exemplificative images of HEK cells co-transfected with the ptdTOMATO-N1vector and scrambled (*ctrl*) or ICln siRNA (*ICln*). The samples were immunolabelled with an anti-4.1R antibody (*4.1R*) or anti-ICln antibody (*ICln*). Scale bar 20 µm. (B) Western blot of total protein extracts from HEK cells co-transfected with ICln siRNA (*ICln*) or scrambled siRNA (*ctrl*) and the fluorescent tdTomato protein. The histograms show the mean OD value of the ICln signal normalised for the corresponding GAPDH signal (n = 4). The values are percentages of the control. **p<0.01.

To better characterise the physiological role of ICln in regulating 4.1R localisation, we performed ICln knockdown experiments (Fig. 3). siRNA for ICln and control scrambled siRNA (siRNA ctrl) were co-transfected in HEK cells together with the fluorescent protein tdTomato, to identify the cells where ICln was downregulated (Fig. 3A, first row). Both in immunofluorescence (Fig. 3A) and western blot experiments (Fig. 3B), the ICln downregulation in cells transfected with siRNA ICln was clearly evident. It should be noted that, due to different expression levels of the fluorescent protein, the cells with low tdTomato levels are not visible in the images. Endogenous 4.1R protein localized in membrane regions both in cells with low expression levels of ICln, and in cells transfected with the control siRNA. However, we observed in two independent experiments that the 4.1R membrane signal was globally more intense in the siRNA ICln sample.

### ICln inhibits 4.1R interactions with sub-membranous actin

**Figure 4:**
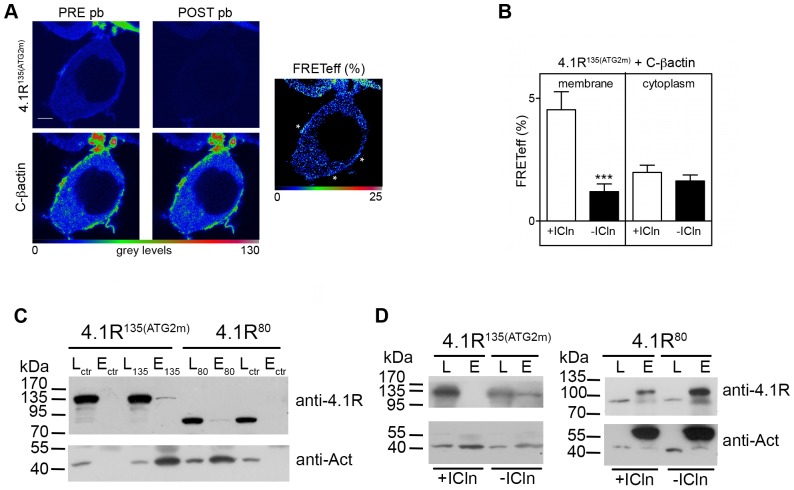
FRET analysis of YFP-tagged 4.1R and CFP/β-actin (C- βactin) interaction. (A) Example of an acceptor photobleaching FRET experiment in HEK cells over-expressing Y-4.1R^135^ and C-βActin. A FRET signal can be seen under the plasma membrane (asterisks). Scale bar 5 µm. FRETeff was measured in ROIs in the plasma membrane or cytoplasm. (B) Mean FRETeff ± SEM in cells over-expressing Y-4.1R^135^, C-βActin and IRES-DsRED (*-ICln*) or Y-4.1R^135^, C-βActin and ICln-IRES-DsRED (*+ICln*). ***p<0.001, -ICln *vs* +ICln. (C, D) Co-immuprecipitation of over-expressed 4.1R^80^ or 4.1R^135^ and actin. (C) HEK cells were transfected with GFP-IRES-4.1R^80/135^, and actin was immunoprecipitated using a goat anti-actin antibody (samples *135* or *80*). Goat Ig-G (bovine alkaline peroxidase) was used as a negative control (*ctr*). (D) C-ICln (*+ICln*) or C (*-ICln*) was co-transfected with 4.1R to investigate the effect of ICln on 4.1R/actin interactions. The Western blots are representative of three (4.1R^135^) or two (4.1R^80^) independent experiments, all with comparable results. The 4.1R (*anti-4.1R*) and actin signals (*anti-Act*) in the cell lysates (*L*) and final eluates (*E*) are shown for all conditions. The additional bands in the 4.1R^80^ Western blot in D are probably residual incompletely denatured (anti-4.1R blot) or denatured (anti-actin blot) antibody chains as they were also recognised by the sole secondary anti-goat antibody.

We investigated whether ICln affects the integrity of the 4.1R/actin/spectrin ternary complex in cell cortical regions. FRET experiments performed to investigate the influence of ICln on 4.1R/actin interactions (Fig. 4) showed that, like the 4.1R^135^ signal, CFP-tagged ß-actin (C-ßactin) localised in the cytoplasm and sub-membrane regions. For this reason, FRET efficiency was measured separately in ROIs of the whole cytoplasm and ROIs of only the thin cytoplasmic layer underlying the plasma membrane (Fig. 4B). This analysis did not include 4.1R^80^ because its FRETeff was no different from that of the control (over-expression of YFP/C-ßactin/IRES-DsRED). The transfected cells showed a low FRET signal that was mainly concentrated in the membrane area. In comparison with the control condition, the cells expressing Y-4.1R^135^/C-ßactin showed a statistically significant FRET in the membrane region (control FRETeff = 0.79±0.41, n = 8; p<0.01) in the absence of ICln over-expression; when ICln was co-expressed, FRETeff decreased to control levels. No significant FRET was observed in the cytoplasm regardless of the presence or absence of ICln (Fig. 4B).

Immunoprecipitation experiments performed upon the over-expression of both 4.1R and C-ICln (or CFP, control) using an anti-actin antibody (Figs. 4C and 4D) confirmed that the presence of ICln tends to reduce the amount of both 4.1R isoforms in actin immunoprecipitate and, in line with the results of the FRET experiments, this effect was more marked in the case of 4.1R^135^.

### 4.1R^80^ (but not 4.1R^135^) enhances the hypotonically induced I_Cl,swell_ current

**Figure 5:**
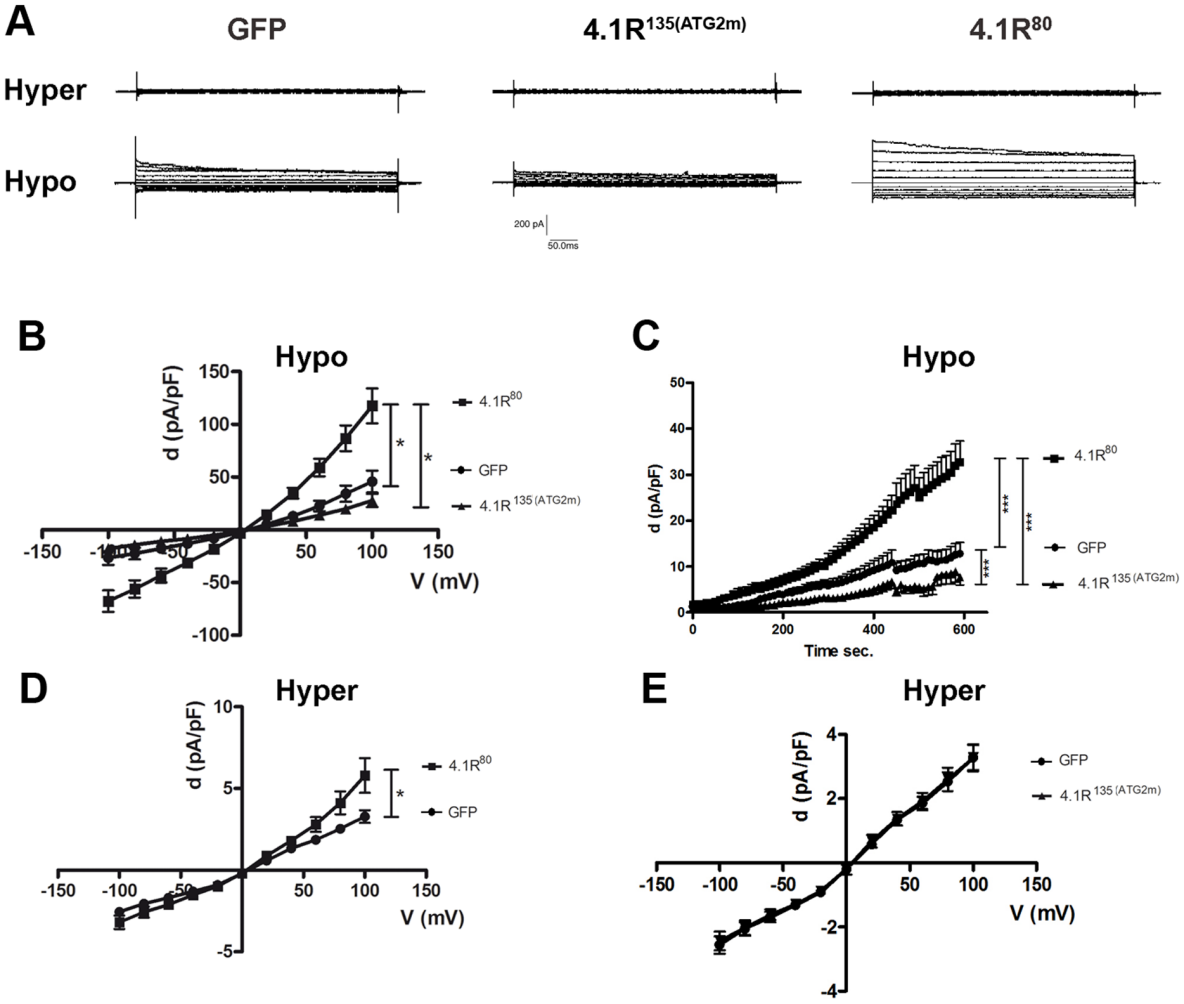
I_Cl,swell_ characterisation in cells over-expressing 4.1R^80/135^. (A) Representative whole-cell traces recorded in control cells (over-expressing GFP) or cells over-expressing the 4.1R^80^ or the 4.1R^135^ protein exposed to hypertonic (*Hyper*) and hypotonic (*Hypo*) extracellular solutions. (B) Relationship between mean current density, *d (pA/pF)*, and membrane voltage, *V (mV)*, in cells over-expressing the indicated proteins and exposed to the hypotonic extracellular solution for 10 min (GFP: n = 22, 4.1R^80^: n = 15, 4.1R^135^: n = 14). (C) Chloride current activation during hypotonic exposure (GFP: n = 24, 4.1R^80^: n = 17, 4.1R^135^: n = 14. (D,E) Relationship between mean current density and membrane voltage in control cells or cells over-expressing the 4.1R^80^ (C) or the 4.1R^135^ protein (D) in the hypertonic extracellular solution (GFP: n = 43, 4.1R^80^: n = 38, 4.1R^135^: n = 27. *p<0.05; ***p<0.001. Two-way ANOVA.

ICln plays a major role in the activation of I_Cl,swell_, a key player of regulatory volume decrease (RVD). Whole-cell patch-clamp experiments were performed to study the functional effects of 4.1R/ICln interactions on the regulation of the I_Cl,swell_ current (Fig. 4). HEK cells transfected with 4.1R^135/80^ were initially maintained in a hypertonic solution (Figs. 5A, 5D and 5E) but, when this was replaced by a hypotonic solution, an outward rectifying current was activated. The reversal potential was not statistically different from 0 mV, as expected for a chloride current, and the current was slightly inactivated at potentials higher than +60 mV (Figs. 5A and 5B). All of these characteristics are in line with those of the swelling-activated I_Cl,swell_ current [54]. The cells transfected with 4.1R^80^ (but not those transfected with 4.1R^135^) showed a statistically significant higher current not only when the hypotonic current was fully activated (Fig. 5B), but also when the basal current was measured under hypertonic conditions (Fig. 5D). Transfection with 4.1R^135^ induced a hypotonically activated current that was not statistically different from the control (Fig. 5B). These findings are in line with the time course of current activation (Fig. 5C), which showed that 4.1R^80^ activated the current more rapidly than both the control and 4.1R^135^, while 4.1R^135^ significantly inhibited current activation in comparison with the control.

### In hypotonically exposed HEK cells, the amount of 4.1R in the plasma membrane decreases and the 4.1R^80^/ICln interaction increases

**Figure 6:**
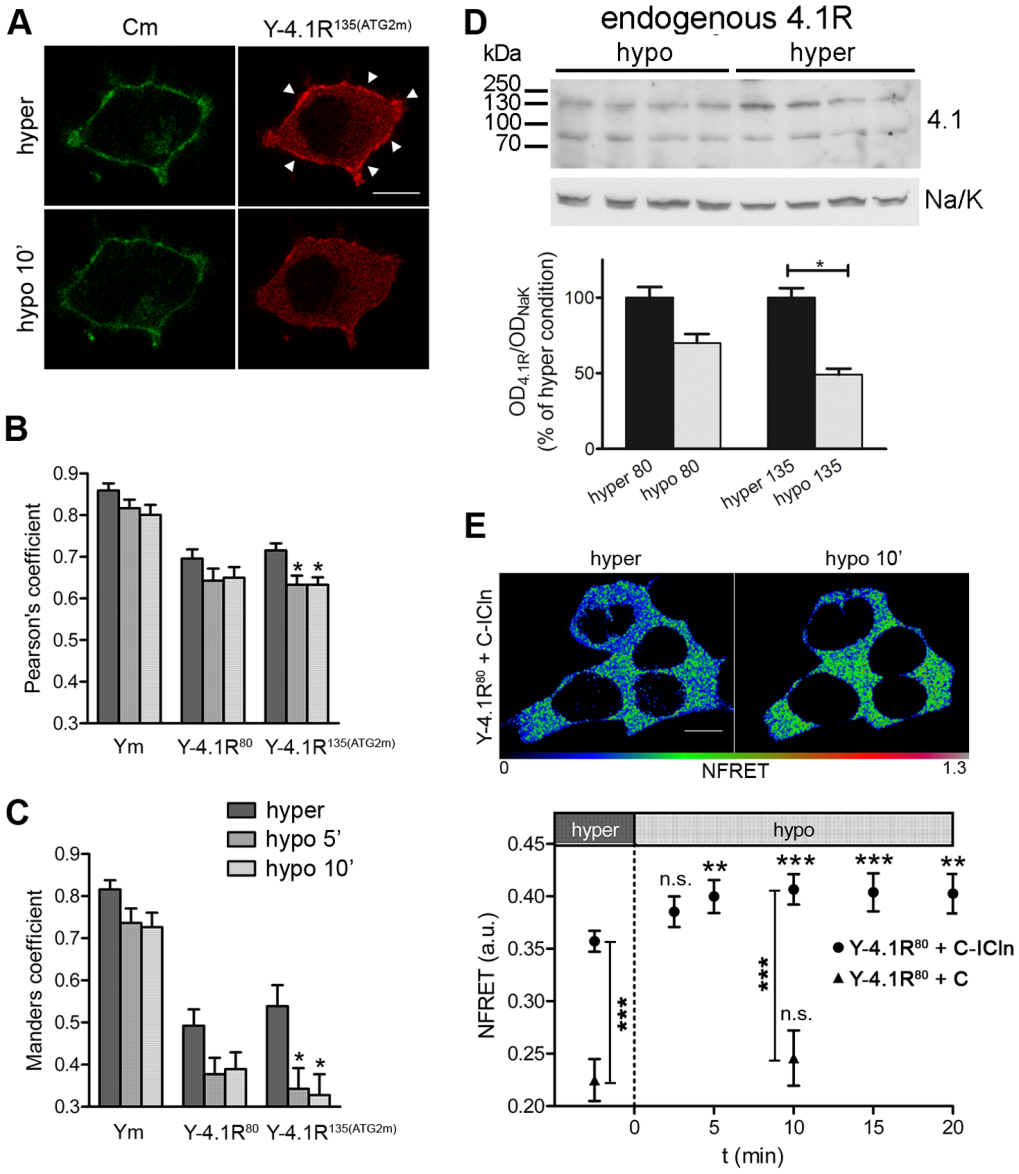
Hypotonic shock affects the membrane localisation of 4.1R, and promotes the 4.1R/ICln interaction. (A) Single confocal plane of a cell expressing the membrane marker CFP-mem (Cm) and the Y-4.1R^135^ protein acquired in the hypertonic (*hyper*) and hypotonic (*hypo 10′*) extracellular solutions. The arrows indicate the membrane regions in which the 4.1R^135^ signal greatly decreases after hypotonic exposure. The mean Pearson (B) and Manders coefficients (C) between Cm and the YFP-tagged proteins indicated in the graphs were calculated from the Z-stacks. The Manders coefficient represents the fraction of the YFP signal overlapping the CFP signal. For each sample, the mean coefficients obtained in hypertonic extracellular solution were compared with the mean coefficients obtained under hypotonic conditions (ANOVA with Dunnett's post-hoc multiple comparison test; *p<0.05). Scale bar 10 µm. (D) Western blot of endogenous 4.1R in total membrane preparations of HEK cells under hypertonic (*hyper*) or hypotonic (*hypo*) conditions. The related graph shows the mean OD of the 4.1R bands normalised for those of the Na/K pump (*OD_4.1R_/OD_NaK_*) (n = 8) used to quantify the changes. The values are percentages of the hypertonic condition. *p<0.05. (E) Representative NFRET images of Y-4.1R^80^/C-ICln expressing cells in the hypertonic extracellular solution or after 10 min exposure to the hypotonic extracellular solution, and the NFRET time course during RVD. In comparison with the hypertonic condition, the NFRET signal in the Y-4.1R^80^/C-ICln expressing cells (but not the control Y-4.1R^80^/C expressing cells) significantly increased after five minutes in the hypotonic extracellular solution (one-way ANOVA with Dunnett's multiple comparison test). The NFRET values of the Y-4.1R^80^/C-ICln expressing cells in the hypertonic extracellular solution were statistically different from those of the control Y-4.1R80/C cells (t-test) after both 0 (hypertonic) and 10 min hypotonic solution exposure. Scale bar 10 µm. ***p<0.001; **p<0.01.

It is known that ICln translocates to membrane regions upon hypotonic challenge [11],[49]. To investigate the dynamics of 4.1R interactions with the plasma membrane during a hypotonic shock, we analysed the co-localisation of the membrane marker CFP-mem (Cm) and the over-expressed YFP-tagged 4.1R (Figs. 6A, 6B and 6C). Pearson and Manders coefficients were measured in the same cells during exposure to the hypertonic extracellular solution, and 5 and 10 minutes after switching to a hypotonic solution (Figs. 6B and 6C). The overall co-localisation (represented by Pearson's coefficient, Fig. 6B) of 4.1R^135^ and Cm significantly decreased in the hypotonic solution and, accordingly, so did the fraction of 4.1R^135^ overlapping Cm (Manders coefficient, Fig. 6C). The control cells were co-transfected with Cm and YFP-mem (Ym).

In line with the co-localisation data, Western blots of the membrane proteins of HEK cells showed a reduction in the amount of membrane-associated 4.1Rs that was significant only in the case of the 135 kDa isoform (Fig. 6D).

These results suggest that a hypotonic shock partially detaches 4.1R proteins from the plasma membrane, and has a greater effect on the 135 kD isoform.

In order to study the 4.1R/ICln interaction upon cell swelling, we performed sensitised emission FRET experiments using Y-4.1R^80^/C-ICln and Y-4.1R^80^/C (Fig. 6E) that allowed us to follow the time-course of the 4.1R^80^/ICln interaction during hypotonic exposure. Analogous experiments could not be performed with the 135 kDa isoform, since no significant FRET signal could be detected with Y-4.1R^135^/C-ICln pair, as previously reported.

The NFRET values (Fig. 6E) indicate that hypotonicity significantly increased the interaction between C-ICln and Y-4.1R^80^, starting after five minutes of hypotonic challenge. The NFRET values in the controls (Y-4.1R^80^/C) were no different from those recorded under hypertonic conditions, thus demonstrating the specificity of the ICln/4.1R^80^ response to hypotonicity (Fig. 6E). These results were confirmed by the acceptor photobleaching experiments in which the FRETeff calculated in the Y-4.1R^80^/C-ICln-expressing cells exposed to the hypertonic extracellular solution significantly increased after 10 min exposure to the hypotonic solution (FRETeff = 11.62±0.86, n = 40, in the hypertonic solution *vs* FRETeff = 15.07±1.17, n = 20, in the hypotonic solution; p<0.05).

### ICln over-expression antagonises the cell spreading and filopodia emission promoted by 4.1R^135^ over-expression

Actin plays an important role in regulating cell spreading [55] and filopodia emission [56],[57], and 4.1 proteins regulate cell adhesion and spreading in mouse keratinocytes [58] and astrocytes [59]. As ICln seemed to affect the membrane and actin binding properties of 4.1R, we investigated whether it also interfered with these functions.

**Figure 7:**
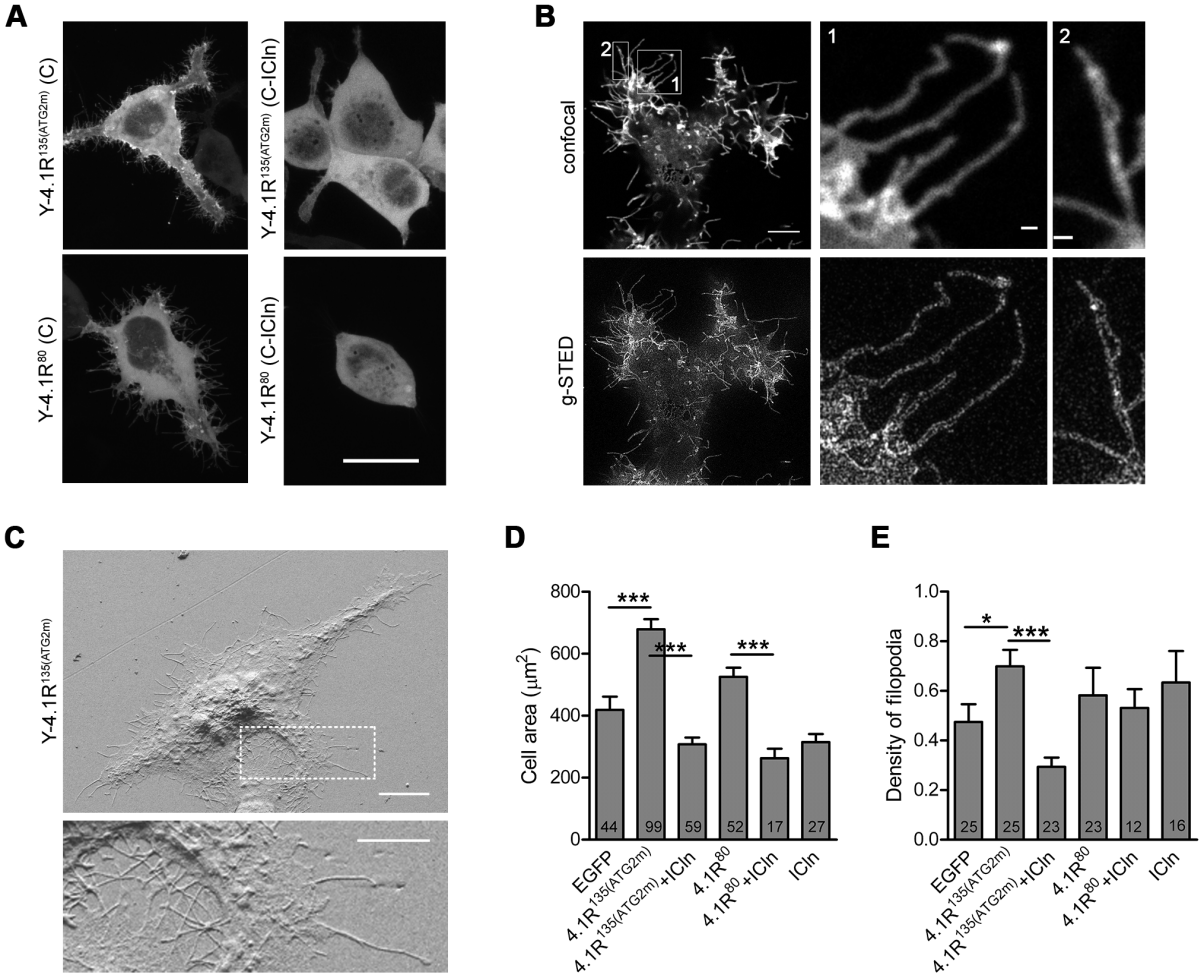
Morphological analysis of HEK cells over-expressing 4.1R and ICln. (A) The maximum projections of a number of optical sections over-expressing the indicated proteins and covering at least half of the cell (z-step size 0.25 µm). Only the YFP channel is shown. Scale bar: 20 µm. (B) The confocal plane of the cell attached to the coverglass acquired using the confocal or gated-STED (g-STED) module of a Leica TCS SP8. The cell over-expressed a membrane marker (YFP-mem) and the 4.1R^135^ protein. Only the YFP signal is shown. Scale bar is 5 µm, and 0.5 µm in 1 and 2. (C) Example of SEM acquisitions of cells transfected with GFP-IRES-4.1R^135^; the lower image is the magnified view of the inset, and makes it possible to see the mesh of cellular filopodia. Scale bar 10 µm (5 µm in the inset). (D) The histogram shows the mean cell area ± SEM (µm^2^) calculated from the SEM images. (E) The mean density of filopodia per cell (the number of filopodia per cell/cell perimeter (µm)). The numbers in the bars represent the number of analysed cells from three independent experiments. *p<0.05; ***p<0.001.

A qualitative evaluation of confocal Z-stack acquisitions indicated that the cells transfected with YFP-tagged 4.1R were larger and that 4.1R induced the growth of an increased number of filopodia, a phenotype that was more pronounced with the over-expression of 4.1R^135^ (Fig. 7A). This phenotype was completely reverted when C-ICln was co-expressed in HEK cells (Fig. 7A). A correlative-light scanning electron microscopy (CLEM) protocol was established in order to measure the cell area and the number and length of the filopodia (Fig. 7C). We used high-resolution electron microscopy because the thickness of filopodia is below the resolution limit of conventional confocal microscopy [57], as confirmed by super-resolution gated-stimulated emission depletion (g-STED) microscopy (Fig. 6B) [60]. Moreover, the detachment of fluorescent 4.1R from the plasma membrane caused by the presence of ICln (Fig. 2) could lead to an underestimate of the number of filopodia when measured by means of standard confocal microscopy (Fig. 7A).

The SEM analysis (Fig. 7D) confirmed that 4.1R^135^ over-expression induced a significant increase in cell surface area, but co-expression with ICln reverted this phenotype. The over-expression of 4.1R^80^ induced a smaller increase that was not statistically different from that of EGFP-expressing cells, but still significantly greater than that measured when ICln was co-expressed.

We also considered the density of the filopodia protruding from the cell profile: i.e. the number of filopodia per cell/cell perimeter (Fig. 7E). In comparison with the control EGFP-expressing cells, the over-expression of 4.1R^135^ induced a significant increase in filopodia density, an effect that was once again reverted by the co-expression of ICln.

The over-expression of 4.1R^80^ did not significantly affect filopodia density, thus suggesting that the two isoforms play a similar but not identical role in dynamically regulating the cortical cytoskeleton. No significant difference in the length of the protrusions could be detected (not shown).

## Discussion

ICln interactions have so far only been reported with 4.1R^80^ variants or single 4.1R domains [8],[22],[61]. Our co-immunoprecipitation results show that ICln interacts with both the 80 and 135 kDa isoforms of native and over-expressed chimeric 4.1R. The FRET experiments demonstrated the direct interaction between ICln and 4.1R^80^, although the co-immunoprecipitation experiments clearly indicated interactions with both the chimeric variants. This apparent incongruity may have been due to the unfavourable and rigid orientation of the fluorophore dipoles in the complex, or the small Förster radius (R_0_) of the CFP/YFP FRET pair (4.72 nm) [62].

One of the main effects of ICln co-expression was a change in the subcellular localisation of both 4.1R proteins. In co-expression with C-ICln both 4.1R proteins were mislocalized: 4.1R binding to the membrane and to the cortical actin cytoskeleton was inhibited and the cytoplasmic pool was increased, as shown in the immunofluorescence images. No variation in the total amount of 4.1R was detected, supporting the hypothesis that the reduction of the membrane pool was not a consequence of protein degradation or of a change in global expression levels. It is feasible that the effects of ICln binding under physiological condition are less dramatic, but it is anyway likely that ICln is one of the factors that negatively affect 4.1R membrane localization, an effect that could be artificially emphasized, but not artificially created, by ICln over-expression. The qualitative evaluation of 4.1R localisation in cells with downregulated ICln is in accordance with such a physiological role of ICln.

One important observation concerning the mechanism by which ICln inhibits the membrane association of 4.1R is that ICln interacts directly with the FERM domain, which is crucial for the association itself and the target of complex regulation [39],[40]. ICln binds to its C-lobe, which also binds to the cell adhesion molecule CD44, phospholipid phosphatidylserine [63] and, together with lobe A, forms a binding site for the cytoskeletal adapter protein p55 [53] and the lipid phosphatidylinositol-4,5-bisphosphate (PIP2), which can also influence actin binding [64]. By interacting with this crucial domain, ICln might alter the affinities for other binding partners, thus inhibiting the association of 4.1R with the cortical actin cytoskeleton and greatly affecting its role in the recruitment of a wide range of proteins involved in signalling [65]–[67], adhesion [58],[68] and ion transport [18],[28]–[30].

It is worth mentioning that the C-terminal lobe of the FERM domain is a PIP2 binding PH (pleckstrin homology) domain [69]; ICln binds to it with its unstructured C-terminal half, leaving its N-terminal half (which is also a PH domain) [70],[71] free to interact with other potential partners. The PH domain of ICln does not have the electrostatic surface polarisation characteristic of PIP2-binding [71] PH domains, and so it could radically change the affinity of 4.1R for PIP2 and, consequently, its interaction pattern.

It has already been shown that 4.1R localisation can be regulated by its interaction with other proteins [68], suggesting that the formation of functional protein complexes is essential for proper 4.1R intracellular localisation and function. ICln-4.1R interaction could represent a way of modulating 4.1R function, by favouring the formation of specific protein complexes in specific subcellular compartments of the cell. One of the main functions of 4.1R proteins is their regulation of membrane transport systems. The 4.1R modulation of erythrocyte Cl^-^/HCO_3_^-^ anion exchanger 1 (AE1) has been clearly documented [41],[61],[72], and many other ion channels and transporters have been added to the list more recently [18],[73]. In particular, it has been suggested that 4.1R may be involved in volume regulation as it has been shown that it physiologically down-regulates Na^+^/H^+^ exchange (which is involved in the process of regulatory volume increase, RVI), and that up-regulation of Na^+^/H^+^ exchange is an important contributor to the high cell Na^+^ content of 4.1^−/−^ mouse erythrocytes [18],[29]. Our findings show that 4.1R^80^ can activate ICl_,swell_, which is involved in RVD, thus suggesting that 4.1R may be a crucial factor linking the complex parallel regulation and synchronisation of the transport systems participating in cell volume regulation, which is related to various other cell housekeeping functions such as cell morphology and proliferation [74],[75].

Our data concerning the mechanism by which 4.1R^80^ activates the I_Cl,swell_ current are not conclusive, but it has been previously reported that 4.1R or other 4.1 isoforms have a *direct* effect on Na^+^, Cl^-^, K^+^ and Ca^2+^ currents [30],[73], and that this has important consequences for cardiac pathology [30],7[6] and nerve conduction [77]–[79]. It has also been suggested that the 4.1 proteins may regulate the membrane expression of these transport systems [73],[80]. However, the picture is particularly complex in the case of I_Cl,swell_ because the identity of the channel protein is still debated [81],[82], although the translocation of ICln towards the membrane is considered to be one of the key processes of I_Cl,swell_ activation [11],[49]. The relation between ICln and the channel responsible for I_Cl,swell_ is far from being understood. It has been proposed that it could be one of the molecular components of the channel itself [83], yet not all agree [82],[84] on this hypothesis. Even if reconstitution of pure ICln proteins in artificial bilayers can result in the conduction of an ion current [83],[85], it has been demonstrated that, in mammalian cells, the association of ICln with the membrane is typical of an extrinsic protein rather than an integral protein [86]. Accordingly, it has been proposed that ICln might be a key regulator of a still unknown channel; its translocation towards the membrane area would be necessary to activate the current, maybe through integrin-related pathways [87], and/or by its interaction with subcortical actin cytoskeleton [14]. Along this line of thought, it is possible that ICln translocation could play a role in the reorganization of the actin cytoskeleton by inhibiting the 4.1R bridging function between the plasmalemma and the subcortical actin ring, and this could be a key event for the activation of the channel.

A complex reorganisation of the actin cytoskeleton during hypotonicity has been reported [14],[88]–[90], and it has been proposed that different cell pools of F-actin (cortical, associated with stress fibres, perinuclear) are involved in regulating swelling-activated channels, possibly with different effects [91]. Our data show that ICln co-expression inhibits the association of 4.1R with the membrane, and that its relocation is associated with detachment from the cortical actin cytoskeleton. It is therefore possible that hypotonicity-induced ICln translocation to the sub-membranous region plays a role in the detachment of 4.1R from the membrane and cortical actin cytoskeleton, and that this is one of the steps leading to I_Cl,swell_ activation. A second factor affecting 4.1R membrane affinity during hypotonicity might be calcium as a calcium transient is a common early event in RVD signalling [92]-[93]. The fact that the membrane association of 4.1R^135^ seems to be more affected by hypotonicity may reflect its greater sensitivity to calcium signalling [35].

These events could participate in the rearrangement of the sub-cortical actin cytoskeleton that accompanies the activation of I_Cl,swell_ and coincides with increased interaction between ICln and actin [14]. The complex of ICln and 4.1R (and maybe other partners) seems to restrict the presence of both proteins to the cytosol, thus reducing their abundance in other sub-cellular pools and possibly modulating 4.1R function.

The fact that the over-expression of 4.1R^80^, but not 4.1R^135^, results in the activation of the current was unexpected but it could suggest that the ratio between the two 4.1R isoforms in the membrane area is a key factor for the activation of the current and ICln could play a role in this process. Others have previously reported differences in the functions and behaviour of the various 4.1R isoforms, such as their binding affinities for membrane proteins [35], and it is likely that the HP region plays a critical role in conferring specific functions to each isoform. It is possible that regulating the ratio between 4.1R^80^ and 4.1R^135^ (rather than the absolute quantity of individual proteins) in the membrane area is crucial for the correct functioning of the sensing and signalling events linking hypotonicity to RVD and for the activation of the I_Cl,swell_ current. In accordance, during RVD we observed (by western blot) a trend to the decreasement of the 4.1R^135^/4.1R^80^ ratio in total membrane preparations (in hypertonic condition: 4.1R^135^/4.1R^80^ = 1.79±0,32, n = 11, in hypotonic condition: 4.1R^135^/4.1R^80^ = 1,18±0,18, n = 12, p = 0.098), even if the result was not significant. This observation agrees well with the co-localisation and western blot data indicating that the hypotonicity induced detachment from the membrane is more pronounced for 4.1R^135^ rather than 4.1R^80^. 4.1R^80^ over-expression could mimic such a condition and could be responsible for the increased I_Cl,swell_ current we measured in hypertonic conditions.

In any case, the emerging picture is that the two isoforms can have different functions and different effects on cell physiology. On the basis of our data, 4.1R^135^ seems to be the main isoform involved in regulating cell adhesion and filopodia emission; it is possible that, by maximising these consequences on cell morphology, its over-expression interferes with hypotonicity-related signalling. I_Cl,swell_ activation has previously been related to actin cytoskeleton stiffness [94],[95], and it is feasible that, by inducing a change in actin cytoskeleton architecture, the change in morphology maximised by 4.1R^135^ negatively affects or at least slows down I_Cl,swell_ activation. The fact that ICln, which usually causes activation of the current [96], can inhibit all 4.1R^135^- related changes in morphology is consistent with the hypothesis that this functional specialisation of 4.1R^135^ may be critical for I_Cl,swell_ activation.

Our findings indicate that cell spreading and the number of filopodia are the microscopic cell features mainly affected by 4.1R over-expression, and that ICln can revert these 4.1R-related effects. It is reasonable to hypothesise that this phenotype is related to the role of 4.1R in organising the actin cytoskeleton [56],[57] by directly binding to F-actin [25], and/or as a consequence of its interaction with integrins at focal adhesions [58], thus leading to the subsequent activation of a signalling pathway that leads to actin reorganisation. It is conceivable that ICln binding interferes with this process by inhibiting the association of 4.1R with the plasma membrane cytoskeleton. Interestingly, it has been demonstrated that ICln can bind integrins in platelets [87],[97], thus supporting the hypothesis that an integrin-activated pathway may be involved in important cell functions such as cell migration, invasion, survival and proliferation [98]–[100]. An alternative or additional possibility is that PKC might as well be involved in the cross-talk between 4.1R and ICln. The PH domain of ICln interacts with and can be phosphorylated by PKC *in vitro* [71]. PKC has also been reported to modulate swelling activated currents [101]–[103] and is one of the modulators of 4.1R binding to the membrane and the spectrin-actin [39],[41] complex, with a significant impact on membrane mechanical stability [39] and deformability [104] and on the regulation of transport systems.

Our findings confirm that 4.1R plays a role in regulating cell spreading. They also show that the co-expression of ICln can revert the observed effects of 4.1R, thus confirming the hypothesis that it acts as a modulator of 4.1R functions and affects the regulation of a variety of membrane channels/transporters, the organisation of signalling systems and the processes of cell division, migration and differentiation.
